# Comparative Study of Hydrochemical Classification Based on Different Hierarchical Cluster Analysis Methods

**DOI:** 10.3390/ijerph17249515

**Published:** 2020-12-18

**Authors:** Jianwei Bu, Wei Liu, Zhao Pan, Kang Ling

**Affiliations:** 1 School of Environmental Studies, China University of Geosciences, No. 68 Jincheng Street, Wuhan 430078, China; jwbu@cug.edu.cn (J.B.); panzhao@cug.edu.cn (Z.P.); 1201810326@cug.edu.cn (K.L.); 2 Technology Innovation Center of Geo-Environmental Restoration, Ministry of Natural Resources, No. 388 Lumo Road, Wuhan 430074, China; 3 Institute of Geological Survey, China University of Geosciences, No. 388 Lumo Road, Wuhan 430074, China

**Keywords:** groundwater leakage, hydrochemical classification, multivariate statistics, hierarchical cluster analysis, Bayi Tunnel

## Abstract

Traditional methods for hydrochemical analyses are effective but less diversified, and are constrained to limited objects and conditions. Given their poor accuracy and reliability, they are often used in complement or combined with other methods to solve practical problems. Cluster analysis is a multivariate statistical technique that extracts useful information from complex data. It provides new ideas and approaches to hydrogeochemical analysis, especially for groundwater hydrochemical classification. Hierarchical cluster analysis is the most widely used method in cluster analysis. This study compared the advantages and disadvantages of six hierarchical cluster analysis methods and analyzed their objects, conditions, and scope of application. The six methods are: The single linkage, complete linkage, median linkage, centroid linkage, average linkage (including between-group linkage and within-group linkage), and Ward’s minimum-variance. Results showed that single linkage and complete linkage are unsuitable for complex practical conditions. Median and centroid linkages likely cause reversals in dendrograms. Average linkage is generally suitable for classification tasks with multiple samples and big data. However, Ward’s minimum-variance achieved better results for fewer samples and variables.

## 1. Introduction

Traditional methods for graphical analysis of hydrochemical data include Piper (trilinear) diagrams, scatter plots, quadrilateral diagrams, rhombus diagrams, triangle diagrams, Schuka Lev classification, Broski classification, Kurllov’s (KypmoBa) formula, etc. [1,2,3,4,5]. Studies relying on one aforementioned method or measure may be susceptible to limited and biased results. For example, the classification of water samples using Piper diagrams tend to be vague and ineffective as it only plots a few major anions and cations [6,7]. The Schuka Lev classification has clear indices (for chemical constituents in groundwater) and a subjective predetermined threshold in milliequivalents (mEq) for ions. Therefore, this method obscures the fuzziness in water quality to some extent, and the variation of water quality is not detailed enough in classification results [8,9,10,11].

In recent years, cluster analysis (CA) and other multivariate statistical methods have been increasingly used in the classification of foundations. They can effectively extract useful information from complex datasets, and provide a reasonable and efficient approach to the study of chemical characteristics of groundwater [12,13]. The main factors affecting the hydrochemical field can be effectively identified using information regarding major ionic and nonionic components of groundwater that are extracted through multivariate statistical methods, which may further facilitate the understanding of the formation mechanism in the hydrochemical field [7,14,15,16,17,18,19]. Furthermore, clustering methods provide comprehensive analysis of the hydrochemical properties and improve the rationality in hydrochemical analysis by showing the sources of recharge, hydraulic relations, transport laws of groundwater, and the interaction characteristics between groundwater and its surrounding environment to a certain extent [20,21,22].

Moreover, CA covers many topics and is flexible. There are many theories and techniques related to CA, which may be applied to various objects and conditions. If the selected technique is unsuitable for a task, characterization of the nature and internal laws of data will be difficult, and may produce results that deviate from reality and the original intention of research. Therefore, core issues that need to be urgently addressed are: (a) Selection of one or several clustering methods for analysis under specific conditions; (b) comparing the advantages and disadvantages of various methods; (c) approximation of actual object compositions and the reflection of the objective laws of data; (d) achieving the optimal process and results through CA.

Therefore, in this study we performed a CA on 19 groups of leakage water samples collected from the Bayi Tunnel in Chongqing (municipality directly under the Central Government) to investigate the internal relationship between the sample data using six hierarchical cluster analysis (HCA) methods, i.e., single linkage, complete linkage, median linkage, centroid linkage, average linkage (including between-groups and within-groups linkage), and Ward’s minimum-variance. In addition, this study compared the advantages and disadvantages of the aforementioned methods and analyzed their objects, conditions, and scope of application.

## 2. Materials and Methods

### 2.1. General Setting of the Study Area

The Bayi Tunnel is located in between the Lianglukou Subdistrict and the Shangqingsi Subdistrict of Yuzhong District in Chongqing, Southwestern China. The entrance of the Bayi Tunnel is located in Jianxinpo, and the exit is at the southeast of the Chongqing Municipal Facilities Administration Bureau. This tunnel passes beneath the Chongqing Emergency Medical Center (CEMC), Chongqing Sports Bureau, and Lines 1 and 3 (Jianxinpo Tunnel) of the Chongqing Rail Transit. This tunnel was constructed in 1984, surrounded by roads in all directions. There is convenient daily traffic in its surrounding areas with dense flows of people and vehicles. It is an important tunnel in the Chongqing traffic hub. However, this tunnel has incurred water leakage and has other issues, partly because of the long service life, and partly because of intense human activities and complex natural conditions in its surrounding areas.

The soil in the study area is mainly composed of Quaternary gray brown clay and gray purple silty sand, mixed with gravel, with good hydraulic conductivity. The outcropping strata are fluvial and lacustrine sedimentary rocks, mainly composed of Jurassic fine sand and silty mudstone. The weathering fracture depth is generally 0.2–1.5 m. The groundwater is mainly distributed in the pores of Quaternary loose layer and weathered fissures of bedrock, which is mainly recharged by precipitation.

### 2.2. Sample Collections

After a rainfall event, a total of 19 water sample sets were collected: One sample set of underground sewer water (USW) from CEMC above the Bayi Tunnel; one set of precipitation (rain) samples from the atmosphere near the tunnel periphery; one sample set of the bedrock fissure water (BFW) and a set of pumping pipeline water (PPW) from superjacent Jianxinpo Tunnel; fifteen leakage water sample sets were collected from the Bayi Tunnel. Three sets of the fifteen were collected from the drain hole in the lining (at 272 m) of the Bayi Tunnel on three consecutive days. Twelve sets were collected on four consecutive days from three leakage points of the tunnel lining, at 327.5, 347, and 355 m, respectively.

Polyethylene bottles with 50-mL capacity were used as sample containers. The bottles were cleaned with distilled water before sampling and then rinsed 2 to 3 times with the water sample to be taken. Each sample set comprised two portions: A sample for cation analysis, to which dilute nitric acid (HNO_3_) was added until its pH was less than 2; and the other sample for anion analysis, which was unprocessed. The sampling process was in line with the relevant specifications and requirements in the Guidance of Collection and Preservation of Groundwater Sample for Quality Control (DZ/T 0064.2-93).

### 2.3. Chemical Analyses

HCO_3_^-^ was measured in the field using a simple titration device with an analysis precision of 0.03 mmol/L (1.83 mg/L). The pH, temperature, and electrical conductivity (EC) measurements were conducted in-field using a Hanna HI8733 portable conductivity meter and Hanna HI8242 portable pH/mV meter, with the analysis precisions of 0.01 (pH), 0.1 °C (temperature), and 1 µs/cm (EC).

Water samples were sent to the State Key Laboratory of Biogeology and Environmental Geology in China University of Geosciences (Wuhan) for cation and anion analyses in one week after the rainfall event. Cations were measured using inductively coupled plasma optical emission spectrometry (ICP-OES, IRIS Intrepid II XSP, Thermo Fisher Scientific, Waltham, MA USA) with a precision of 1 × 10^−3^ mg/L, and anion analysis was performed using an ion chromatograph (IC, DX-120, Dionex, Sunnyvale, CA USA) with a precision of 0.01 mg/L (Table 1).

### 2.4. Data Quality Assurance

National reference materials (NRM) of China, GSBZ 50017-90 (202158 pH = 4.12, 202164 pH = 7.35, 202160 pH = 9.04), GBW(E) 130285 (EC = 12.88 ms/cm), GBW(E) 130415 (EC = 1000 µs/cm), and GBW(E) 130416 (EC = 100 µs/cm) have been applied for Hanna HI8242 and HI8733 calibrations. GSBZ 50017-90, GSB 04-1720-2004, GSB 04-1733-2004, GSB 04-1735-2004 (a), GSB 04-1738-2004, GSB 04-1770-2004, GSB 04-1771-2004, GSB 04-1772-2004, and GSB 04-1773-2004 (a) have been utilized for measuring pH, Ca^2+^, K^+^, Mg^2+^, Na^+^, Cl^−^, F^−^, NO_3_^−^, and SO_4_^2−^, respectively. Six concentration gradients of NRM ranging from 1 to 200 mg/L (1, 5, 10, 50, 100, 200 mg/L) have been established as calibration standards for cation measurement. By contrast, eight concentration gradients of NRM ranging from 0.1 to 200 mg/L (0.1, 0.5, 2, 5, 10, 50, 100, 200 mg/L) have been selected as calibration standards to measure anions.

WS 02 and WS 08 represent USW from CEMC and rain from tunnel periphery, respectively. Due to the particularity of these two samples, NO_3_^−^ from WS 02, together with Mg^2+^ from WS 08, have not been detected. Affected by sampling time (before and after the rainfall), Ca^2+^ and Mg^2+^ have detected no data at the same time from WS 07 and WS 14. In order to excavate the internal relationship between different water sample types, as well as the temporal transforming pattern from the same water sample type, these four water samples with missing value(s) were reserved for CA. Because the contents of these variables are lower than the detection limits, 0 was introduced to replace the no data in CA.

The charge-balance error (CBE) was within ±5%, as the percentage relative total of the cation–anion difference was calculated on the sums from each water sample (Table 1). All analyses yielded analytical errors <5% and external precision of known–unknown analytical standards. To better ensure the quality of raw data, EC was also processed and calculated to compare with total dissolved solids (TDS) [23,24,25]. Unary linear regression equation of TDS(*y*) versus EC(*x*), *y* = *0.7117x*, was extracted with *R^2^* = 0.9906. All procedures of sampling, preservation, and transportation to the laboratory were strictly conducted in accordance with standard methods [26].

### 2.5. Cluster Analysis (CA)

#### 2.5.1. Concept

CA is a multivariate statistical method that gradually classifies samples based on their similarity. It regards the samples as points in a multidimensional space, and the similarity between points are indicated using statistics [13,27]. Objects with a high degree of similarity are classified into a small cluster, while those with a low degree of similarity are classified into a large cluster. This classification continues until all data objects are classified. In CA, a data set is divided into several clusters, and the objects in the same cluster have a higher degree of similarity than those in other clusters [12,28,29]. CA is seen as a typical combinatorial optimization problem, which is described by the following mathematical model.

In a given set of pattern samples {***X***}, there are *n* samples and *k* classes of patterns {*S_j_*, j = 1,2, …, k}. Each sample contains *m* variables. The set ***X*** can be expressed by a matrix as:

$$X\;=\;(x_{1},x_{2},\text{…},x_{n})=\;\left(\begin{array}{l} x_{11}\;\;x_{12}\;\;\ldots \;\;x_{1n}\\ x_{21}\;\;x_{22}\;\;\ldots \;\;x_{2n}\\ \ldots \;\;\ldots \;\;\ldots \;\;\ldots \\ x_{m1}\;x_{m2}\;\;\ldots \;\;x_{\mathrm{mn}} \end{array}\right)$$

Each column of ***X*** is a sample, where *x*_1*i*_, *x*_2*i*_, …, *x*_m*i*_ denote the first, second, …, *m*-th variable of the *i*-th sample. To classify samples, the minimum distance between each sample and its cluster center is taken as the similarity or distance metric, and its objective function is:

$$T\;=\;\min\sum_{j=1}^{k}\sum_{X\in S_{i}}^{}\left\|X\right.-\left.m_{j}\right\|\;m_{j}=\frac{1}{\sum_{i=1}^{n}y_{\mathrm{ij}}}\sum_{i=1}^{n}y_{\mathrm{ij}}X_{i}$$

where *k* is the number of clusters; *m_j_* denotes the mean vector of the *j*-th sample (*S_j_*); $\sum_{i=1}^{n}y_{\mathrm{ij}}$ = 1, implying that the sample *i* is only assigned to a cluster center. The classification rule is that if *i* is assigned to *j*-th cluster center, then *y_ij_* = 1; or else, *y_ij_* = 0.

#### 2.5.2. Hierarchical Cluster Analysis

Existing clustering algorithms mainly include hierarchical clustering, partitioning, density-based clustering, grid-based clustering, model-based clustering, and fuzzy clustering. In particular, hierarchical clustering consists of hierarchical decomposition of a given set of data objects. Each object is initially regarded as an individual cluster. Then, objects with the shortest distance are joined into a new cluster until all are joined together in one large cluster.

Depending on the definition of the nearest (neighbor) distance and the recursion equation for clustering, hierarchical clustering can be subdivided into single linkage, complete linkage, median linkage, centroid linkage, average linkage, and Ward’s minimum-variance [30]. At present, hierarchical clustering is the most widely used clustering method. The related calculation and analysis modules have been integrated into many statistical analysis software packages or systems, such as SPSS, SAS, and S-PLUS, so that the users can directly invoke relevant functions.

##### Single Linkage

**Figure 1:**
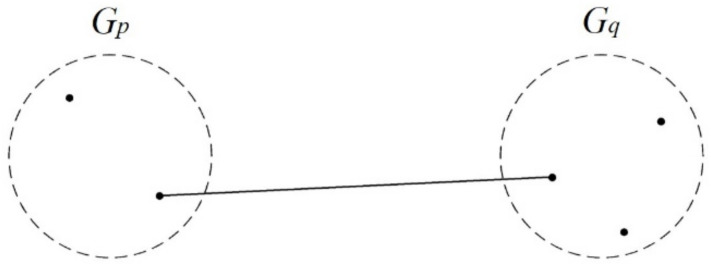
Conceptual diagram of the single linkage.

In single-linkage clustering, the two closest clusters are joined into a new cluster, and the shortest distance between members (in different clusters) is the distance between the new cluster and another cluster. Two clusters with the shortest distance are joined until one large cluster remains (Figure 1).

Let the distance between *x_i_* and *x_j_*, i.e., *d*(*x_i_,* *x_j_*), be represented as *d_ij_*. Let *G_p_* and *G_q_* denote two clusters containing *n_p_* and *n_q_* objects, respectively. *D*(*G_p_*, *G_q_*) or *D_pq_* represent the distance between clusters *G_p_* and *G_q_*. Let *G_r_* = {*G_p_*} represent the new cluster that *G_p_* and *G_q_* join into.

The distance between clusters *G_p_* and *G_q_* is defined as the distance between their closest members, which is referred to as the shortest distance. It is calculated as:

*D*(*G_p_*, *G_q_*) = min{***d**_ij_*|*i*∈*G_p_*, *j*∈*G_q_*, *p ≠ q*}

After *G_p_* and *G_q_* are joined into a new cluster *G_r_*, the distance between *G_r_* and another cluster G*_k_* (*k ≠ p, q*) is calculated based on the single-linkage clustering using the formula below:

*D*(*G_r_*, *G_k_*) = min{***d****_ij_*|*i*∈*G_r_*, *j*∈*G_k_*}

=min{min{***d****_ij_*|*i*∈*G_p_*, *j*∈*G_k_*}, min{***d****_ij_*|*i*∈*G_q_*, *j*∈*G_k_*}}

=min{*D*(*G_p_*, *G_k_*), *D*(*G_q_*, *G_k_*)}

##### Complete Linkage

**Figure 2:**
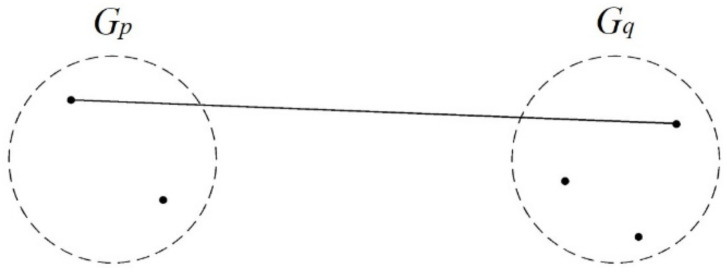
Conceptual diagram of the complete linkage.

This method joins two closest clusters into a new cluster and takes the longest distance between its members as the distance between the new cluster and another cluster. Among the farthest-apart members, two clusters that have the shortest distance are joined until all members are in the same cluster (Figure 2).

The distance between clusters *G_p_* and *G_q_* is defined as the distance between their farthest-apart members, which is referred to as the longest distance. It is calculated as:

*D*(*G_p_*, *G_q_*)=max{***d**_ij_*|*i*∈*G_p_*, *j*∈*G_q_*, *p* ≠ *q*}

After *G_p_* and *G_q_* are joined into a new cluster *G_r_*, the distance between *G_r_* and another cluster G*_k_* (*k ≠ p, q*) is calculated using the complete-linkage clustering through the following formula:

*D*(*G_r_*, *G_k_*) = max{***d****_ij_*|*i*∈*G_r_*, *j*∈*G_k_*}

=max{max{***d****_ij_*|*i*∈*G_p_*, *j*∈*G_k_*}, max{***d****_ij_*|*i*∈*G_q_*, *j*∈*G_k_*}}

=max{*D*(*G_p_*, *G_k_*), *D*(*G_q_*, *G_k_*)}

##### Median Linkage

**Figure 3:**
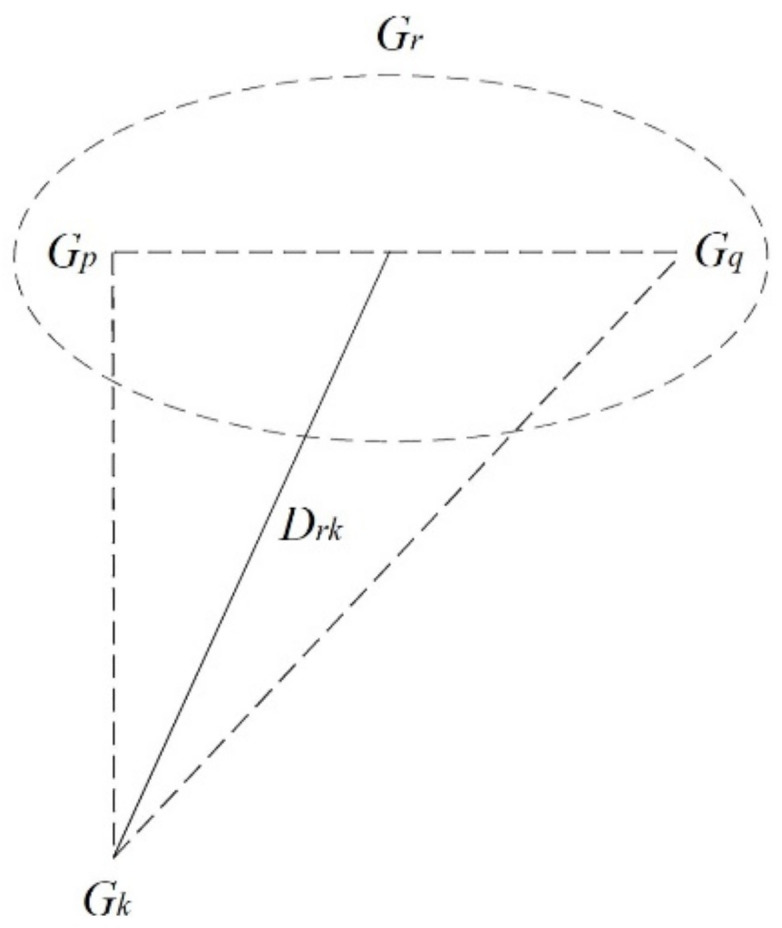
Conceptual diagram of the median linkage.

The shortest and longest distances in single and complete linkages represent two extremes in distance measurement. In contrast, median linkage uses an approach that falls within the shortest and complete linkages for calculating the distance between clusters (Figure 3).

After *G_p_* and *G_q_* join into a new cluster *G_r_*, the distance between *G_r_* and another cluster G*_k_* (*k ≠ p, q*) is calculated based on median linkage using the equation below:

$$D^{2}(G_{r},\;G_{k})\;=\;\frac{1}{2}(D_{pk}^{2}+D_{qk}^{2})+\beta D_{pq}^{2}\left(-\frac{1}{4}\leq \beta \leq 0\right)$$

where *β* is often set to $\beta =-\frac{1}{4}$. Here, *D_rk_* is the midsegment across the side *D_pq_* of the triangle formed by *D_pk_*, *D_qk_*, and *D_pq_*.

##### Centroid Linkage

**Figure 4:**
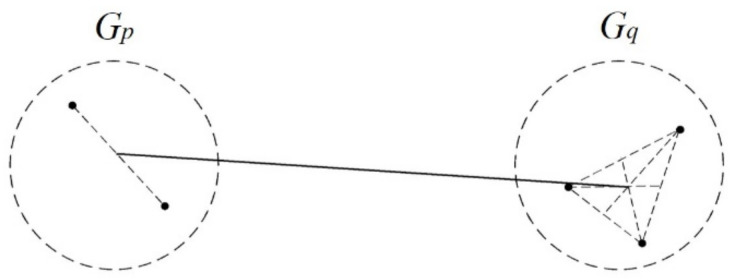
Conceptual diagram of the centroid linkage.

From a physical perspective, representing a cluster with its centroid is more reasonable. In centroid linkage, the distance between the centroids of two clusters is used to measure the distance between clusters. The distance between clusters is defined as the distance between their centroids. In object classification, the centroid for a cluster is considered to be the mean value of objects in that cluster (Figure 4).

After *G_p_* and *G_q_* are joined into a new cluster *G_r_*, they contain *n_p_*, *n_q_*, and *n_r_* (*n_r_* = *n_p_* + *n_q_*) objects, respectively. Their centroids are denoted as ${\bar{X}}^{\left(p\right)}$, ${\bar{X}}^{\left(q\right)}$, and ${\bar{X}}^{\left(r\right)}$, respectively. We obtain:

$${\bar{X}}^{\left(r\right)}\;=\;\frac{1}{n_{r}}(n_{p}{\bar{X}}^{\left(p\right)}\;+\;n_{q}{\bar{X}}^{\left(q\right)})$$

The distance between *G_r_* and another cluster *G_k_*(*k ≠ p, q*) is:

$$D^{2}(G_{r},\;G_{k})\;=\;{\left({\bar{X}}^{\left(k\right)}-{\bar{X}}^{\left(r\right)}\right)}^{T}\left({\bar{X}}^{\left(k\right)}-{\bar{X}}^{\left(r\right)}\right)\;=\frac{n_{p}}{n_{r}}D_{pk}^{2}+\frac{n_{q}}{n_{r}}D_{qk}^{2}-\frac{n_{p}n_{q}}{n_{r}^{2}}D_{pq}^{2}$$

##### Average Linkage

Average linkage considers the average distance between members in two clusters, which can be further subdivided into two types: Between-groups linkage and within-groups linkage. When calculating the distance between clusters, between-groups linkage considers the average distance between members in different clusters, while within-groups linkage considers the distance between all members.

The distance between *G_p_* and *G_q_* is defined as the average distance between their member pairs, which is referred to as the average distance between clusters. It is calculated as:

$$D^{2}(G_{p},\;G_{q})\;=\;\frac{1}{n_{p}n_{q}}\sum_{i\in G_{p}}\sum_{j\in G_{q}}d_{ij}^{2}$$

The distance between the new cluster *G_r_* and another cluster *G_k_* (*k ≠ p, q*) is calculated as:

$$D^{2}(G_{r},\;G_{k})\;=\;\frac{n_{p}}{n_{r}}D_{pk}^{2}+\frac{n_{q}}{n_{r}}D_{qk}^{2}$$

where *n_r_* = *n_p_* + *n_q_*.

a. Between-groups linkage

**Figure 5:**
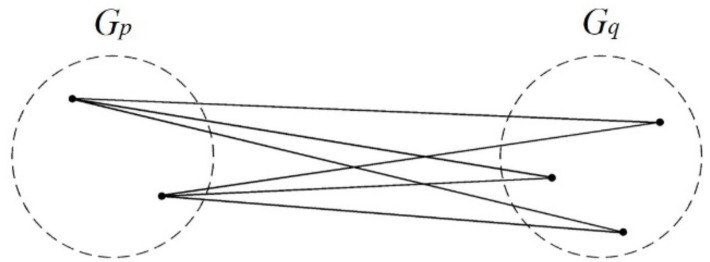
Conceptual diagram of the between-groups linkage.

This method defines the distance between two clusters as the average distance between their member pairs, and the two members are from different clusters. At each step, two clusters with the shortest average distance are merged until all members are joined into a large cluster (Figure 5). In other words, the average distance between each member pairs of two clusters is the shortest after they merge into a new cluster using between-groups linkage.

b. Within-groups linkage

**Figure 6:**
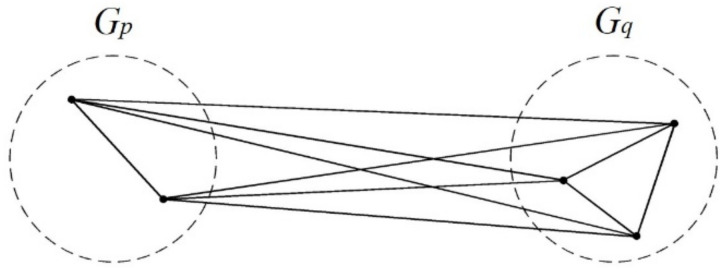
Conceptual diagram of the within-groups linkage.

This method defines the distance between two clusters as the average distance between any two members of the clusters, including the distance between any two members, irrespective of the cluster. At each step, two clusters with the shortest average distance are merged until all members are joined into a large cluster (Figure 6). This means that after two clusters merge into a new cluster, the average distance between their members in the new cluster is minimized.

##### Ward’s Minimum-Variance

This method is based on the analysis of variance (ANOVA). For the correct classification, the ANOVA results show small within-groups sum of squares and large between-groups sum of squares.

Assuming that *n* samples are categorized into *k* groups, the *i*-th sample in the cluster *G_t_* is denoted as $X_{i}^{\left(t\right)}$, and *n_t_* represents the number of samples in *G_t_*. Let the centroid of the cluster be ${\bar{X}}^{\left(t\right)}$. Then, the sum of squares within *G_t_* is:

$$S_{t}=\sum_{i=1}^{n_{t}}{\left(X_{i}^{{}^{\left(t\right)}}-{\bar{X}}^{\left(t\right)}\right)}^{T}(X_{i}^{{}^{\left(t\right)}}-{\bar{X}}^{\left(t\right)})$$

The total sum of squares for *k* groups is:

$$S=\sum_{t=1}^{k}S_{t}\;=\sum_{t=1}^{k}\sum_{i=1}^{n_{t}}{\left(X_{i}^{{}^{\left(t\right)}}-{\bar{X}}^{\left(t\right)}\right)}^{T}(X_{i}^{{}^{\left(t\right)}}-{\bar{X}}^{\left(t\right)})$$

In Ward’s minimum-variance method, *n* samples are initially considered as separate clusters. Each time two clusters merge, the number of clusters decreases by one, and *S* increases. At each step, the two clusters are merged, resulting in the least increase of *S*, until all samples are joined into the same cluster.

The distance between *G_p_* and *G_q_* is defined as the sum of squares between the two clusters:

$$D^{2}(G_{p},\;G_{q})\;=\;S_{r}-S_{p}-S_{q}$$

where *G_r_* = {*G_p_*, *G_q_*}. The distance between the new cluster *G_r_* and another cluster *G_k_* (*k ≠ p, q*) is calculated as:

$$D^{2}(G_{r},\;G_{k})\;=\;\frac{n_{k}+n_{p}}{n_{r}+n_{k}}D_{kp}^{2}+\frac{n_{k}+n_{q}}{n_{r}+n_{k}}D_{kq}^{2}-\frac{n_{k}}{n_{r}+n_{k}}D_{pq}^{2}$$

#### 2.5.3. Data Standardization

Because the observed values of each variable of samples have different orders of magnitude and measurement units, data transformations are necessary to obtain dimensionless data to avoid inefficient classification and improve the classification accuracy. After utilizing Z-scores to standardize raw data, the mean value of the transformed data was 0, and the standard deviation was 1 (standard normal distribution) in this study (Table 2):

We have $x_{i}{{}_{j}}^{*}=\frac{x_{i}{}_{j}-\bar{x_{i}}}{S_{i}}$ (i = 1, 2, …, m; j = 1, 2, …, n)

where $\bar{x_{i}}=\frac{1}{n}\sum_{j=1}^{n}x_{i}{}_{j}$; $S_{i}=\sqrt{\frac{1}{n-1}\sum_{j=1}^{n}(x_{i}{}_{j}-\bar{x_{i}}{)}^{2}}$, (i = 1, 2, …, m).

#### 2.5.4. Euclidean Distance

The distance is often used as a quantitative indicator for the degree of similarity between samples. Each sample is regarded as a point in an *m*-dimensional space. By defining a certain distance between points in *m*-dimensional space, we can classify the closer points to the same cluster and farther ones into different clusters. This study uses Euclidean distance (Table 3):

$$d(x_{i},x_{j})=\sqrt{{\sum_{k=1}^{m}\left(x_{ki}-x_{kj}\right)}^{2}}$$

All calculations and classification results in this study are obtained using SPSS (IBM, Amonk, NY, USA).

## 3. Results

### 3.1. Single Linkage Method

**Figure 7:**
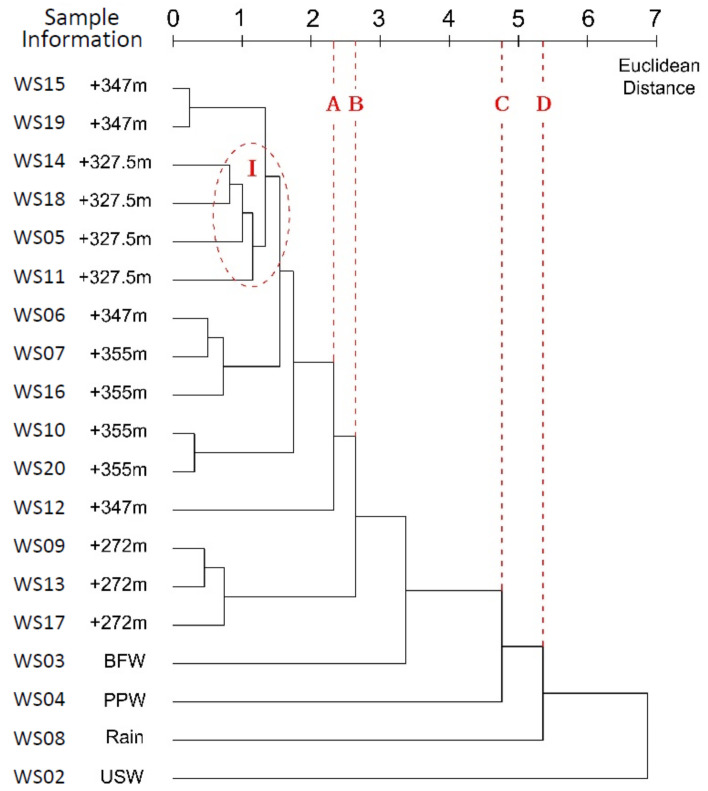
Dendrogram of data through single-linkage clustering.

According to Figure 7, if a line is drawn (Line A) at the Euclidean distance of 2.33, 6 clusters are made: Water leaked from the Bayi Tunnel, running water from the drain hole, BFW and PPW from the Jianxinpo Tunnel, and rain and USW from the CEMC. At the distance of 4.76, three clusters were formed, while only one large cluster existed at the distance of 6.871.

If a line (Line B) was drawn at the distance of 2.643, leaked water from the tunnel and the running water from the tunnel drain hole would join into a cluster, indicating a correlation between the two. However, these two types of water samples were distinguished at a distance less than 2.643, showing difference between the running water through the tunnel drainage system and the water in the hydrochemical process during leakage.

### 3.2. Complete Linkage Method

**Figure 8:**
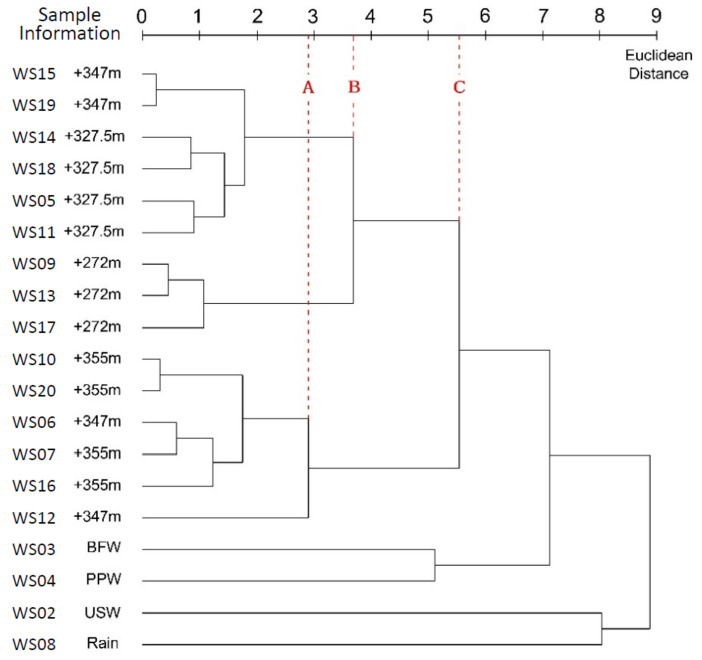
Dendrogram of data through complete-linkage clustering.

According to Figure 8, if a line (Line B) is drawn at the Euclidean distance of 3.691, six clusters are made, four clusters at the distance of 5.551 (Line C), while only one large cluster at the distance of 8.881. At a distance of 5.551, water leaked from the tunnel and the running water from the tunnel drain hole were joined, indicating a certain correlation between water leaked from different parts of the tunnel. At the distance of 2.9 (Line A), water leaked from the tunnel was clearly divided into three types: (a) The running water from the tunnel drain hole at +272 m; (b) water leaked near the point at +327.5 m; and (c) water leaked near the point at +355 m. The gradual changes in hydrochemistry of water samples with different sampling locations were reflected in the clustering process and the dendrogram.

### 3.3. Median Linkage Method

**Figure 9:**
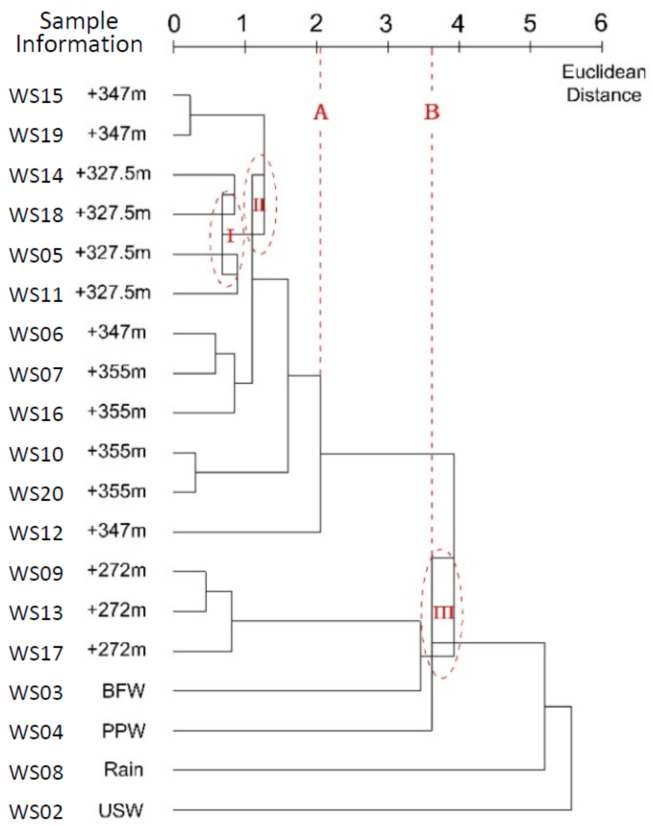
Dendrogram of data through median linkage.

Single linkage underestimated the distance between clusters, while complete linkage exaggerated the distance between clusters. Median linkage provided an approach that fell within the scope of these two linkages. According to Figure 9, if a line (Line A) is drawn at a Euclidean distance of 2.062, six clusters are formed: Water leaked from the Bayi Tunnel; the running water from the drain hole in the tunnel; BFW and PPW from the Jianxinpo Tunnel; and rain and USW from the CEMC. At a distance of 3.614 (Line B), three clusters were formed: One cluster included the water leaked from the tunnel, the running water from the tunnel drain hole, and BFW and PPW from the Jianxinpo Tunnel. One cluster only included rain, while another cluster only included USW. This result suggests the composition difference between rain from the atmosphere and USW of the CEMC. In contrast, there was only one large cluster at a distance of 5.567.

### 3.4. Centroid Linkage Method

From a physical perspective, it is more reasonable to represent a cluster with its centroid. In centroid linkage, the distance between the centroids of two clusters is used to represent the distance between clusters. In object classification, the centroid for a cluster is considered to be the mean of objects in that cluster.

**Figure 10:**
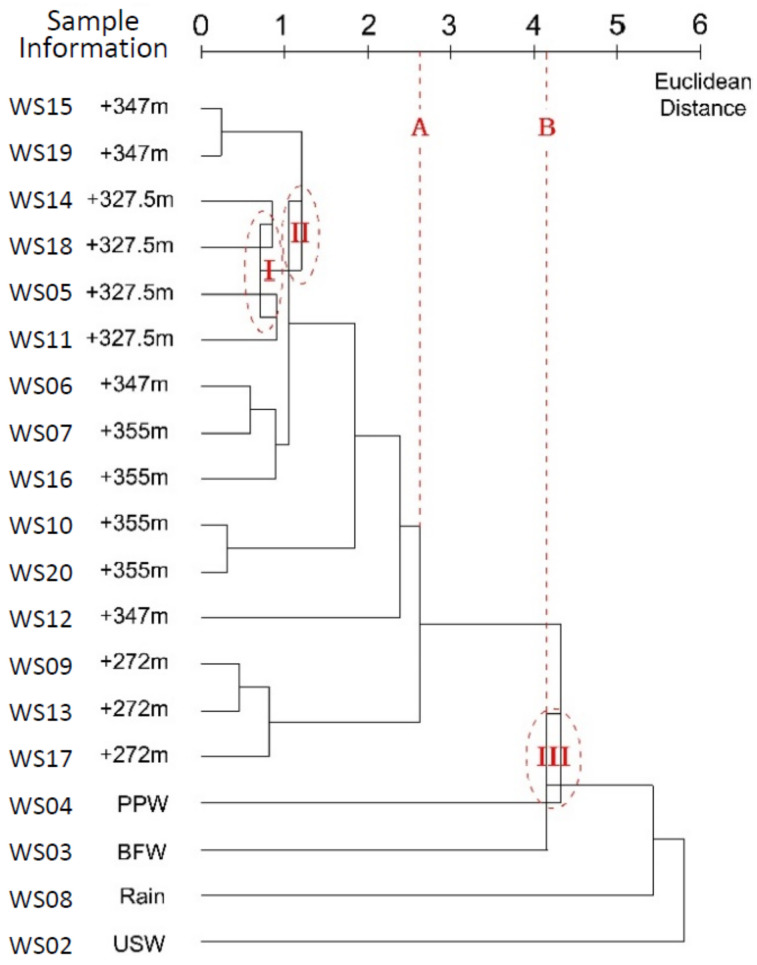
Dendrogram of data through the centroid linkage.

According to Figure 10, if a line (Line A) is drawn at a Euclidean distance of 2.626, five clusters are formed: Water leaked and the running water from the drain hole in Bayi Tunnel; BFW from the Jianxinpo Tunnel; PPW from the Jianxinpo Tunnel; rain; and USW from the CEMC. In median linkage, water leakage from the tunnel and the running water from the drain hole were considered as two different types of water. This differentiation reflects a slight difference between median linkage and centroid linkage, though they were joined at a different distance in centroid linkage.

At a distance of 4.163 (Line B), three clusters were formed, which is consistent with the classification results of median linkage. Specifically, one cluster included water leaked from the tunnel, the running water from the drain hole in the tunnel, and BFW and PPW from Jianxinpo Tunnel. One cluster only included rain, while another cluster only included USW of the CEMC. The above results show the similarities between centroid linkage and median linkage. In contrast, there was only one large cluster at a distance of 5.793.

### 3.5. Average Linkage Method

#### 3.5.1. Between-Groups Linkage

**Figure 11:**
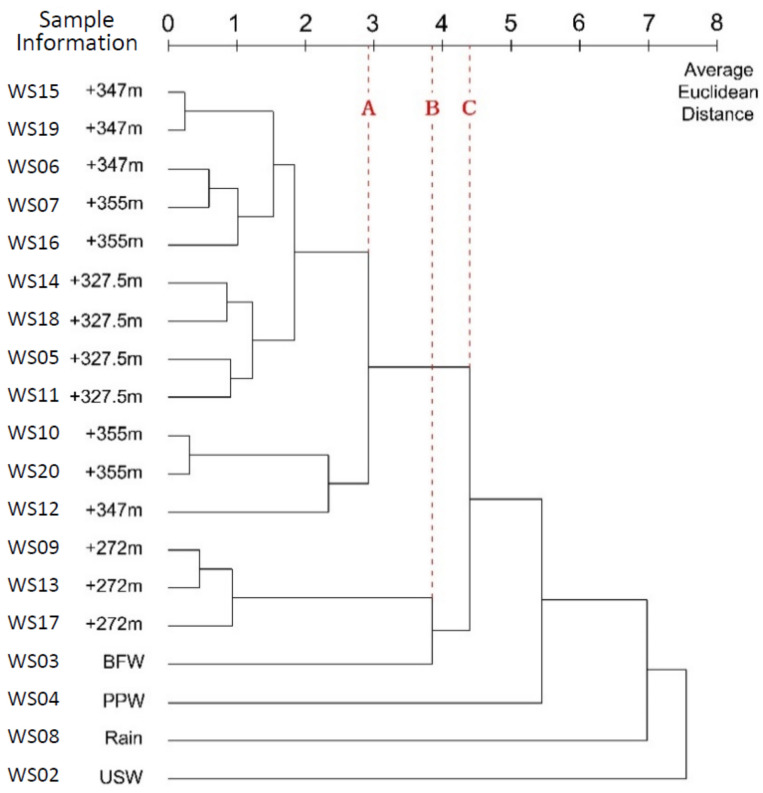
Dendrogram of data through between-groups linkage.

According to Figure 11, if a line (Line A) is drawn at an average Euclidean distance of 2.916, the 19 samples will be categorized into six clusters: Water leaked from the Bayi Tunnel; the running water from the drain hole in the tunnel; BFW from the Jianxinpo Tunnel; PPW from the Jianxinpo Tunnel; rain; and USW from the CEMC. At a distance of 4.401 (Line C), 4 clusters were formed. One cluster included the water leaked from the tunnel, the running water from the drain hole in the tunnel, and the BFW from the Jianxinpo Tunnel. One cluster included the PPW from the Jianxinpo Tunnel, while another cluster included rain and USW from the CEMC. In contrast, only one large cluster existed at a distance of 7.553.

#### 3.5.2. Within-Groups Linkage

**Figure 12:**
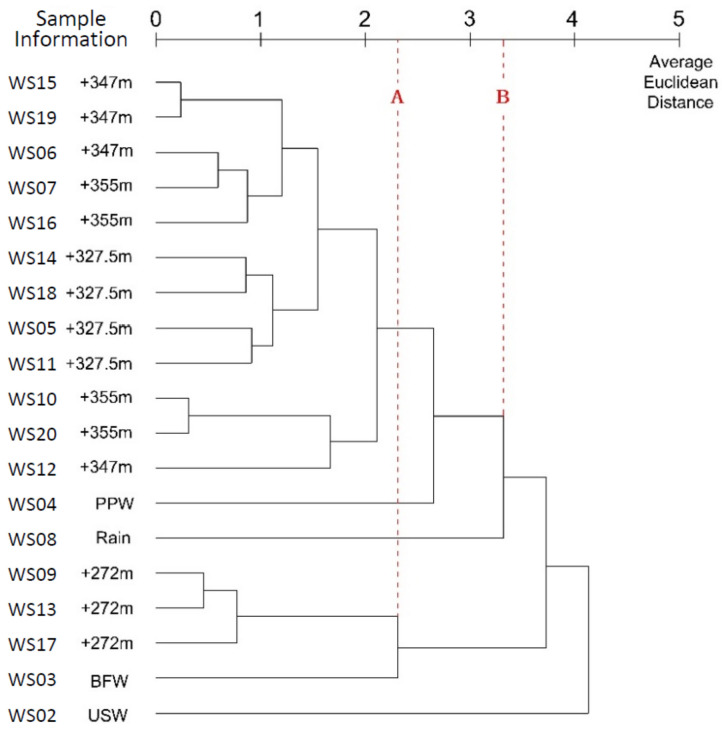
Dendrogram of data through the within-groups linkage.

According to the dendrogram in Figure 12, 19 groups of samples were classified into three clusters at a distance of 3.316 (Line B). One cluster included the water leaked from the tunnel, PPW from Jianxinpo Tunnel, and rain. This classification suggests that the water loss from leakage in the Jianxinpo Tunnel and the Bayi Tunnel may be replenished through rainfall. One cluster included the running water from the drain hole in the Bayi Tunnel and the BFW from the Jianxinpo Tunnel. This indicates a connection between the two and a certain hydraulic relation in rock mass between the two tunnels. Another cluster only included the USW from the CEMC. It showed poor connection with other types of water samples, which were observed in results with other methods. This is because USW is human sewage or wastewater with complex composition, which is completely different from the composition of water samples that are naturally produced.

### 3.6. Ward’s Minimum-Variance Method

**Figure 13:**
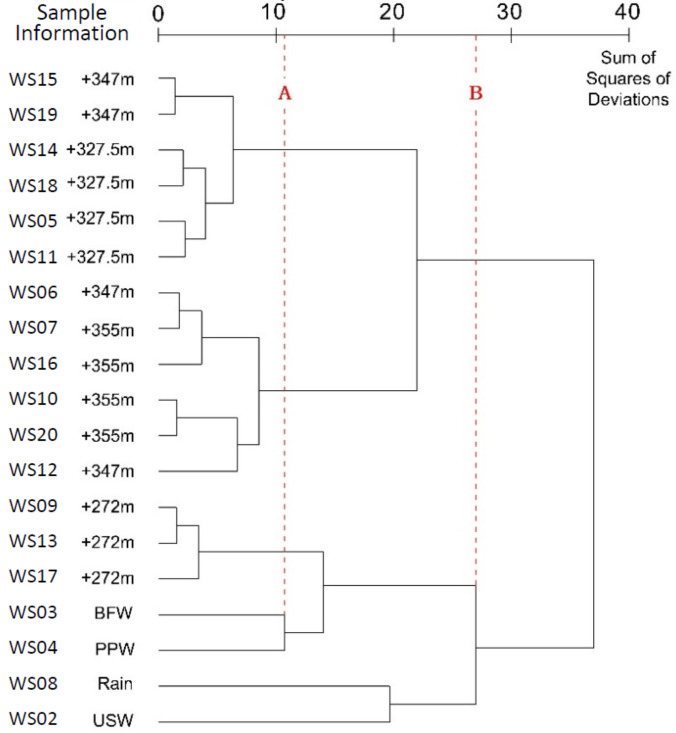
Dendrogram of data through the Ward’s minimum-variance method.

According to the dendrogram in Figure 13, if a line (Line B) is drawn at the sum of squares of 27.467, the 19 groups of water samples will be classified into two large clusters: A cluster with only the water leaked from Bayi Tunnel, and the other cluster with other water samples. The 19 groups of water samples could be further classified into six clusters at the sum of squares of 10.837 (Line A): Water leaked near the point at +327.5 m; water leaked near the point at +355 m; the running water from the drain hole; BFW and PPW from the Jianxinpo Tunnel; rain and USW from the CEMC.

## 4. Discussion

### 4.1. Single Linkage Method

In Figure 7, the leaked water from the tunnel only joins BFW from the Jianxinpo Tunnel and rain at distances of 4.76 (Line C) and 5.357 (Line D), respectively. This indicates the absence of a close direct correlation and the significant effects of delayed or lagged rainfall. The water leaked from the tunnel finally joined USW at the late stage of clustering, showing composition differences between water samples. It is inferred that the pipeline was unlikely to be the source of water leak.

The single linkage method is simple and easy to use, which reflects the basic idea of hierarchical clustering in the most intuitive way. The obtained clustering results were consistent with the water samples determined at the initial sample collection stage. This finding suggests that without external influence and interference, single-linkage clustering showed great performance in data classification and characterization, and could be used to produce relatively clear and accurate clustering results.

However, owing to its inherent limitations in methodology, the closest distance was selected at each step. Sometimes in a long period of clustering, these shortest distances were very close. This may result in little differentiation in clustering steps (see the joint marked by “I” in Figure 7), which may further intervene with the clustering process and classification mapping.

Moreover, the dendrogram of data through this method is in a ladder-like shape and shows an extended-chain structure, implying that links are inevitable. Therefore, the internal connections among samples may be obscured to some extent. This is because the distance between clusters was the shortest. After the two clusters were joined into a new cluster, the distance between the new cluster and any other clusters was shortened, so it was easier to form a large cluster, and most samples were joined in the same cluster. In addition, existing literature shows that single linkage is significantly affected by outliers [31], which limits its application in processing complex data.

### 4.2. Complete Linkage Method

BFW and PPW from the Jianxinpo Tunnel, USW of the emergency center, and rain appeared to have greater distance from the water leaked from the tunnel, suggesting a gradual weakening of the relationship. A relatively strong relationship between the water from the tunnel drainage system and water leaked in the tunnel could be inferred. However, their chemical composition was still slightly different because of different paths and seepage time.

In the complete linkage method, the distance between clusters was defined as the longest distance between the clusters, which made adjustments and improvement on the basis of single linkage. It avoided the inevitable generation of links in single linkage. After the two clusters merged, their distance to other clusters was considered to be the distance from one of the two clusters that had the largest distance. This method increased the distance between the merged cluster and other clusters, and avoided the inevitable generation of links and a ladder-like pattern. Compared to single linkage, the horizontal axis of the dendrogram was extended and covered a larger range in the complete linkage, which produced a more refined clustering result. Objects were further classified into small clusters, and could be used to better characterize the data. Despite its advantages, relevant literature shows that this method may result in many clusters and data distorted by outliers, when dealing with data having large dispersions [32].

### 4.3. Median Linkage Method

The sample order was the same in dendrograms of median linkage and single linkage. Furthermore, results showed the integrity of water leaks in the tunnel and a connection between the running water from the drain hole and BFW. This information was unclear in the previous results, indicating that this method is better in portraying certain details.

Nevertheless, anomalies were detected during clustering. As shown in steps 9, 11, and 16 in the dendrogram below, the distance for merging was less than the distance in the previous step. Reversals (labeled as “I, II, and III”) were observed, which resulted in crossing lines and closed links. Given the non-monotonicity of median linkage, the clustering results were often unsatisfactory, and it was difficult to track links using the dendrogram [33]. Therefore, this method is rarely used.

### 4.4. Centroid Linkage Method

In centroid linkage, the sample order in a dendrogram was similar to that of single linkage and median linkage. In addition, its clustering process was similar to that of median linkage, especially with samples of water leakage in small clusters. The centroid linkage differed from median linkage in the middle stage of clustering. The median linkage strengthened the relationship between the running water from the drain hole and PPW from the Jianxinpo Tunnel, which was stronger than the connection with the water leaked from the tunnel. However, the water leaked from tunnel and the running water from the tunnel drain hole were considered to be within the same large cluster, so their correlation with BFW from the Jianxinpo Tunnel was poor.

Three anomalies were observed during the centroid linkage clustering where the distance for merging was less than the distance in the previous step. Similarly, anomalies occurred in steps 9, 11, and 16. This is the exact same order of anomalies in median linkage clustering. Even the first outlier (0.786) was the same. These small statistical values would inevitably cause partial reversals in the dendrogram. The three abnormal distances for merging were 0.786, 1.053, and 4.163, which correspond to closed links labeled as “I, II, and III (Figure 10)” in the dendrogram, respectively.

Centroid linkage requires the Euclidean distance. Each time the two clusters were merged, the cluster centroids had to be recalculated. Therefore, this method is less affected by outliers. While clusters were well represented by centroid linkage, reversals were likely to occur in dendrograms as the distance between clusters did not follow a monotonous increasing trend [27,34]. It is difficult to track links in the dendrogram, and the symbols may change frequently. In addition, it may involve complex calculation, which further limits its applications.

### 4.5. Average Linkage Method

#### 4.5.1. Between-Groups Linkage

According to the clustering results with between-groups linkage, the relationship between the running water from the drain hole and BFW from the Jianxinpo Tunnel was strengthened, though such an effect only occurred in step 14 of merging at the average Euclidean distance of 3.844 (Line B). Based on the clustering analysis with the aforementioned methods, it can be inferred that BFW from the Jianxinpo Tunnel had a closer connection with the water leaked and the running water in the Bayi Tunnel than other water samples.

As shown in the dendrogram below, between-group linkage resolved the issue in single and complete linkages where the distance between clusters was easily affected by extreme values. It defined the distance between two small clusters as the average distance between all sample pairs, which utilized the distance information of all sample pairs [35].

#### 4.5.2. Within-Groups Linkage

Similar to between-groups linkage, the results of clustering with within-groups linkage showed a correlation between BFW from the Jianxinpo Tunnel and the running water from the drain hole in the Bayi Tunnel at an average Euclidean distance of 2.309 (Line A). During the within-group linkage clustering, the correlation between PPW from the Jianxinpo Tunnel, rain, and the water leaked from Bayi Tunnel was improved, which was not observed in the clustering results with the aforementioned methods.

The within-groups linkage method calculates the average distance of sample pairs, including the pairs between small clusters and pairs within the same cluster. Compared to between-group linkage, it considers the similarity of objects within the same cluster in each clustering step. This method makes use of the known information and considers all samples and individuals. As shown in the dendrogram below, this clustering method achieves good clustering results and has wide applications in practice.

### 4.6. Ward’s Minimum-Variance Method

Compared to the aforementioned methods, the results and effects of clustering with Ward’s minimum-variance method were most consistent with the original type of sample collections. This is because the method required the distance between samples in Euclidean distance, and the distance between two clusters was significantly affected by the number of samples in the two clusters. Therefore, the two clusters tended to be far apart, making it difficult to merge the two. Nevertheless, this approach often met the actual requirements for practical clustering. Therefore, this method performs well in differentiating objects and shows great resistance to interferences. The results of classification using this method were less affected by outliers. Its dendrogram was often clearly structured, straightforward, accurate, and well represented the classification results.

In dealing with the classification of small samples, Ward’s minimum-variance method makes full use of the sample information to explore the internal connection in the data. In the event of little differentiation in samples, this method enlarges the differences between clusters and captures the essential attributes of clusters, thereby providing accurate and reliable classification results [27,36]. In the past, the application of Ward’s minimum-variance method was restricted by the complicated calculations. With the growth of computational technology, it is no longer a great challenge to manage such calculations. Therefore, this method is a very effective clustering method in theory and practice.

### 4.7. Hydrochemical Characteristics

**Figure 14:**
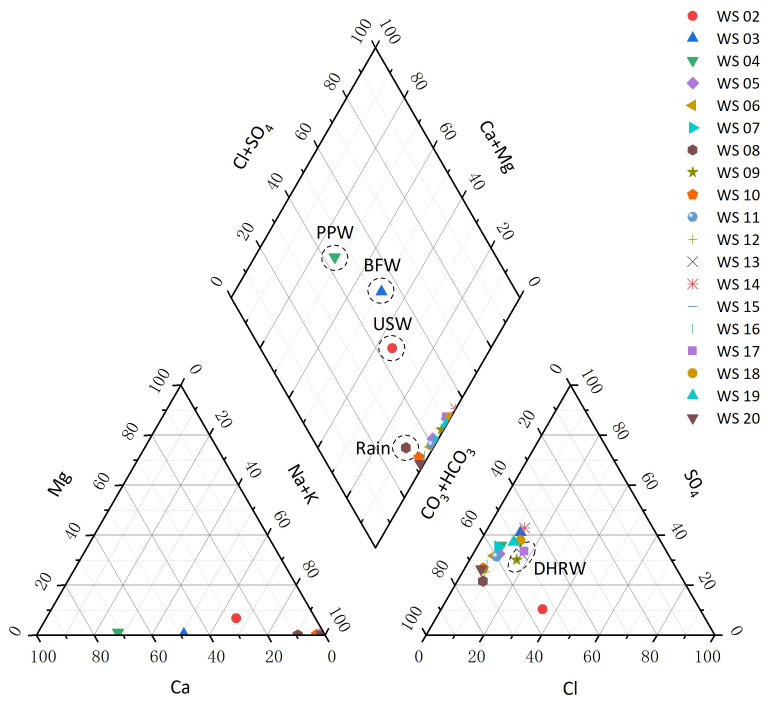
Piper diagram of 19 water samples.

Traditional methods of hydrochemical analysis, Piper trilinear diagram, Schuka Lev classification, and Kurllov’s formula were also conducted to interpret the geneses, connections, and the classifications of these water samples. As shown in Figure 14, Bayi Tunnel has a good aggregation of leakage water, and it is close to the rainfall with time passing by, which shows that the tunnel leakage water is strongly mixed by rainfall, and further shows that the rainfall has an extremely important impact on the leakage water of the tunnel. From different aspects of classification in Table 4, the leakage water types of Bayi Tunnel basically preserved the same, showing significant differences from the rainfall, the CEMC USW, the Jianxinpo Tunnel BFW and PPW, which is consistent with the results of CA. This indicates that the CA results of multivariate statistical methods and the results of traditional hydrochemical analysis had strong comparability and could be mutually verified.

## 5. Conclusions

(1) In the HCA, single linkage was the most basic, comprehensible, and accessible method, which reflected the concept of hierarchical clustering directly. However, it was limited by little differentiations in clustering steps and the inevitable linking tendency (as seen from the ladder-like shapes in dendrograms). Complete linkage adjusted and improved the basis of single linkage. It avoided the inevitable generation of links and ladder-shaped dendrograms. By increasing the distance between clusters for merging, clustering with complete linkage was more refined and data sensitive. However, both single and complete linkage were significantly affected by outliers, and were therefore ineffective when processing data with large dispersions;
(2) Unlike single and complete linkage, median linkage avoided measuring extreme distances, whereas centroid linkage emphasized the representativeness of a cluster. The centroids of clusters had to be recalculated each time after every two clusters merged; therefore, centroid linkage performed more stably when dealing with outliers. However, given the non-monotonicity of these two methods, the distance for merging was likely less than the distance in the previous step, which may have led to reversals, partially closed and crossing links, or other issues in dendrograms. Therefore, these two methods were not recommended;
(3) Average linkage was the default method in the HCA module in SPSS. It included two techniques (i.e., between-group linkage and within-group linkage), and both could make full use of known information. All samples and indicators were considered, and the clustering process was not easily affected by outliers. Average linkage performed well in clustering and was recommended for dealing with a large number of samples, complex variables, and indicators;
(4) Ward’s minimum-variance method could capture and enlarge the differences between clusters that were subtle, hidden, and difficult to identify using other methods, which was conducive to data classification. Using this method, more information could be delivered and expressed, which increased the classification accuracy. For classification tasks with fewer objects and variables, this method could effectively improve the accuracy and classification sensitivity, which could help to explore the essential attributes of data.

## Figures and Tables

**Table 1:** Chemical analyses of water samples (unit: mg/L except pH).

Sample Number	Sampling Location	Water Type	pH	Na^+^	K^+^	Ca^2+^	Mg^2+^	Cl^−^	SO_4_^2−^	CO_3_^2−^	HCO_3_^−^	F^−^	NO_3_^−^	TDS
WS 02	CEMC	USW	7.03	123.396	16.348	58.096	14.251	231.87	68.41	-	357.60	0.31	-	897.484
WS 03	Jianxinpo tunnel	BFW	9.27	93.060	14.991	103.398	0.926	24.35	81.95	83.52	9.57	1.39	67.92	523.827
WS 04	Jianxinpo tunnel	PPW	9.62	77.533	13.630	233.702	3.771	26.33	117.23	153.52	29.30	0.74	6.03	728.329
WS 05	+327.5 m	LW	9.43	225.495	128.598	6.641	0.185	43.92	158.71	271.16	13.16	1.12	4.48	925.866
WS 06	+347 m	LW	8.64	242.497	104.697	2.948	0.252	45.85	190.27	142.34	220.06	1.10	3.64	1038.044
WS 07	+355 m	LW	8.69	233.404	103.904	-	-	44.98	220.18	145.28	211.09	1.11	0.94	1042.903
WS 08	Tunnel periphery	Rain	5.37	6.161	2.555	0.908	-	4.46	10.77	-	34.68	0.08	7.88	71.649
WS 09	+272 m	DHRW	8.72	111.902	11.661	0.848	0.103	44.74	82.59	65.29	81.33	1.28	11.60	445.418
WS 10	+355 m	LW	8.58	261.199	134.796	10.964	0.941	47.43	197.10	94.11	400.66	1.04	5.47	1234.969
WS 11	+327.5 m	LW	8.82	213.104	119.203	2.292	1.194	42.98	154.31	249.98	43.65	1.20	10.63	906.029
WS 12	+347 m	LW	8.66	233.002	98.785	3.634	0.795	58.67	195.06	83.52	429.36	0.24	9.80	1178.830
WS 13	+272 m	DHRW	8.84	120.696	14.640	0.303	0.171	43.67	82.79	88.23	41.26	1.35	15.43	445.471
WS 14	+327.5 m	LW	9.48	212.302	110.803	-	-	41.35	139.92	131.76	13.16	1.21	2.77	718.916
WS 15	+347 m	LW	8.41	218.403	82.868	1.517	0.084	46.87	148.38	54.11	134.55	1.12	2.47	759.789
WS 16	+355 m	LW	8.51	239.996	118.797	3.695	2.016	42.06	170.79	200.57	188.97	1.02	5.73	1042.38
WS 17	+272 m	DHRW	8.73	134.504	15.554	2.054	0.230	43.62	84.86	68.23	56.21	1.29	26.87	470.354
WS 18	+327.5 m	LW	9.14	217.597	98.575	0.305	0.483	40.60	111.51	123.52	17.94	1.03	2.39	670.903
WS 19	+347 m	LW	8.38	230.903	84.268	0.728	0.046	45.88	145.72	52.94	146.51	1.08	2.22	779.646
WS 20	+355 m	LW	8.53	258.095	125.765	4.475	0.411	44.26	197.35	108.82	397.67	1.06	3.96	1221.649

TDS: Total dissolved solids; CEMC: Chongqing Emergency Medical Center; LW: Leakage water; USW: Underground sewer water; BFW: Bedrock fissure water; PPW: Pumping pipeline water; DHRW: Drain hole running water.

**Table 2:** Results of dimensionless standardization of water variables.

Sample Number	pH	Na^+^	K^+^	Ca^2+^	Mg^2+^	Cl^−^	SO_4_^2−^	HCO_3_^−^	F^−^	NO_3_^−^	TDS
WS 02	−1.61573	−0.79248	−1.15515	0.61412	3.96129	3.99725	−1.19004	1.41	−1.80109	−0.65175	0.33212
WS 03	0.73282	−1.20678	−1.18254	1.40611	−0.13248	−0.58216	−0.9467	−0.93993	1.06835	3.76961	−0.87724
WS 04	1.09978	−1.41884	−1.20993	3.68417	0.74037	−0.53847	−0.31265	−0.80671	−0.65863	−0.25922	−0.21535
WS 05	0.90057	0.60173	1.10549	−0.28557	−0.35991	−0.1503	0.43283	−0.91569	0.35099	−0.36012	0.42401
WS 06	0.07229	0.83387	0.62416	−0.35008	−0.34147	−0.10771	1.00003	0.48132	0.29785	−0.4148	0.78706
WS 07	0.12471	0.70961	0.60805	−0.40166	−0.41831	−0.12691	1.53757	0.42075	0.32442	−0.59056	0.80279
WS 08	−3.35617	−2.39341	−1.43287	−0.38578	−0.41831	−1.02108	−2.22594	−0.77038	−2.41218	−0.13879	−2.34078
WS 09	0.15617	−0.94951	−1.2496	−0.38683	−0.38757	−0.13221	−0.9352	−0.4554	0.77609	0.10337	−1.13103
WS 10	0.00938	1.08923	1.23035	−0.21004	−0.12941	−0.07285	1.12278	1.70075	0.13844	−0.29568	1.42445
WS 11	0.26101	0.43241	0.91618	−0.36159	−0.05257	−0.17105	0.35375	−0.70982	0.56354	0.04022	0.35979
WS 12	0.09326	0.70415	0.50514	−0.33818	−0.17243	0.17519	1.08611	1.89453	−1.98707	−0.01381	1.24275
WS 13	0.28198	−0.82934	−1.18959	−0.39636	−0.36606	−0.15582	−0.9316	−0.72595	0.96207	0.35269	−1.13086
WS 14	0.95299	0.42148	0.74701	−0.40166	−0.41831	−0.20701	0.09514	−0.91569	0.59011	−0.47144	−0.24581
WS 15	−0.16886	0.50478	0.18452	−0.37514	−0.39372	−0.0852	0.24718	−0.09605	0.35099	−0.49097	−0.11353
WS 16	−0.06401	0.7178	0.90812	−0.33706	0.20252	−0.19135	0.64993	0.2714	0.0853	−0.27875	0.80111
WS 17	0.16665	−0.6409	−1.17126	−0.36575	−0.34762	−0.15692	−0.8944	−0.62501	0.80266	1.09739	−1.05034
WS 18	0.59652	0.49385	0.5007	−0.39633	−0.27078	−0.22357	−0.41545	−0.88341	0.11187	−0.49617	−0.40123
WS 19	−0.20031	0.67547	0.21271	−0.38893	−0.40294	−0.10705	0.19938	−0.0153	0.24471	−0.50724	−0.04925
WS 20	−0.04304	1.04689	1.04849	−0.32344	−0.2923	−0.1428	1.12727	1.68056	0.19158	−0.39397	1.38134

**Table 3:** Euclidean distance matrix of water samples.

Sample Number	Euclidean Distance																		
	WS02	WS03	WS04	WS05	WS06	WS07	WS08	WS09	WS10	WS11	WS12	WS13	WS14	WS15	WS16	WS17	WS18	WS19	WS20
**WS02**	0	8.881	7.455	7.927	7.435	7.667	8.04	7.283	7.508	7.53	6.871	7.484	7.92	7.167	7.08	7.481	7.491	7.158	7.577
**WS03**	8.881	0	5.119	5.727	6.156	6.42	7.271	4.208	6.959	5.296	7.13	3.953	5.442	5.522	6.017	3.369	5.363	5.631	6.946
**WS04**	7.455	5.119	0	5.349	5.697	5.834	7.09	4.76	6.422	5.306	6.26	4.824	5.212	5.208	5.537	4.925	5.031	5.289	6.428
**WS05**	7.927	5.727	5.349	0	1.841	2.02	7.532	3.663	3.08	0.912	3.968	3.593	0.903	1.74	1.726	3.691	1.405	1.775	3.044
**WS06**	7.435	6.156	5.697	1.841	0	0.593	7.511	3.937	1.565	1.633	2.781	4	2.215	1.443	0.804	4.018	2.397	1.368	1.436
**WS07**	7.667	6.42	5.834	2.02	0.593	0	7.737	4.185	1.728	1.913	2.9	4.252	2.418	1.759	1.218	4.303	2.708	1.727	1.583
**WS08**	8.04	7.271	7.09	7.532	7.511	7.737	0	5.357	8.342	7.091	7.606	5.591	7.129	6.43	7.25	5.624	6.474	6.467	8.229
**WS09**	7.283	4.208	4.76	3.663	3.937	4.185	5.357	0	5.139	3.275	5.371	0.453	2.988	2.716	3.903	1.063	2.674	2.866	5.004
**WS10**	7.508	6.959	6.422	3.08	1.565	1.728	8.342	5.139	0	2.909	2.33	5.239	3.555	2.83	1.745	5.182	3.705	2.717	0.311
**WS11**	7.53	5.296	5.306	0.912	1.633	1.913	7.091	3.275	2.909	0	3.875	3.203	1.176	1.337	1.352	3.215	1.426	1.403	2.869
**WS12**	6.871	7.13	6.26	3.968	2.781	2.9	7.606	5.371	2.33	3.875	0	5.551	4.363	3.54	2.801	5.414	4.217	3.411	2.346
**WS13**	7.484	3.953	4.824	3.593	4	4.252	5.591	0.453	5.239	3.203	5.551	0	2.909	2.79	3.954	0.805	2.643	2.945	5.113
**WS14**	7.92	5.442	5.212	0.903	2.215	2.418	7.129	2.988	3.555	1.176	4.363	2.909	0	1.539	2.155	3.107	0.855	1.634	3.481
**WS15**	7.167	5.522	5.208	1.74	1.443	1.759	6.43	2.716	2.83	1.337	3.54	2.79	1.539	0	1.482	2.908	1.385	0.237	2.697
**WS16**	7.08	6.017	5.537	1.726	0.804	1.218	7.25	3.903	1.745	1.352	2.801	3.954	2.155	1.482	0	3.944	2.201	1.402	1.718
**WS17**	7.481	3.369	4.925	3.691	4.018	4.303	5.624	1.063	5.182	3.215	5.414	0.805	3.107	2.908	3.944	0	2.831	3.041	5.073
**WS18**	7.491	5.363	5.031	1.405	2.397	2.708	6.474	2.674	3.705	1.426	4.217	2.643	0.855	1.385	2.201	2.831	0	1.434	3.63
**WS19**	7.158	5.631	5.289	1.775	1.368	1.727	6.467	2.866	2.717	1.403	3.411	2.945	1.634	0.237	1.402	3.041	1.434	0	2.584
**WS20**	7.577	6.946	6.428	3.044	1.436	1.583	8.229	5.004	0.311	2.869	2.346	5.113	3.481	2.697	1.718	5.073	3.63	2.584	0

**Table 4:** Classifications of traditional hydrochemical analysis methods.

Sample Number	Sampling Location	Schuka Lev Classification		Kurllov’s Formula
WS 08	Tunnel periphery	HCO_3_-(Na+K)	7-A	$M_{0.06}\frac{HCO^{3}{}_{69}SO^{4}{}_{22}}{{\left(Na+K\right)}_{91}}T18.8\;{}^{\circ}C$
WS 02	CEMC	HCO_3_·Cl-(Na+K)·Ca	25-A	$M_{0.87}\frac{HCO^{3}{}_{54}Cl_{35}SO^{4}{}_{10}}{{\left(Na+K\right)}_{66}Ca_{27}}T20.1\;{}^{\circ}C$
WS 03	Jianxinpo Tunnel	SO_4_-(Na+K)·Ca	32-A	$M_{0.33}\frac{SO^{4}{}_{71}Cl_{21}}{{\left(Na+K\right)}_{51}Ca_{49}}T21.5\;{}^{\circ}C$
WS 04		SO_4_-Ca·(Na+K)	32-A	$M_{0.50}\frac{SO^{4}{}_{68}HCO^{3}{}_{17}Cl_{15}}{Ca_{71}{\left(Na+K\right)}_{28}}T20.8\;{}^{\circ}C$
WS 09	+272 m	SO_4_·HCO_3_-(Na+K)	14-A	$M_{0.33}\frac{SO^{4}{}_{40}HCO^{3}{}_{39}Cl_{21}}{{\left(Na+K\right)}_{99}}T21.9\;{}^{\circ}C$
WS 13		SO_4_·Cl·HCO_3_-(Na+K)	21-A	$M_{0.30}\frac{SO^{4}{}_{49}Cl_{26}HCO^{3}{}_{25}}{{\left(Na+K\right)}_{100}}T23.2\;{}^{\circ}C$
WS 17		SO_4_·HCO_3_-(Na+K)	14-A	$M_{0.34}\frac{SO^{4}{}_{46}HCO^{3}{}_{30}Cl_{24}}{{\left(Na+K\right)}_{99}}T23.4\;{}^{\circ}C$
WS 05	+327.5 m	SO_4_-(Na+K)	35-A	$M_{0.58}\frac{SO^{4}{}_{74}Cl_{20}}{{\left(Na+K\right)}_{98}}T22.0\;{}^{\circ}C$
WS 11		SO_4_-(Na+K)	35-A	$M_{0.58}\frac{SO^{4}{}_{64}HCO^{3}{}_{18}Cl_{18}}{{\left(Na+K\right)}_{99}}T22.6\;{}^{\circ}C$
WS 14		SO_4_-(Na+K)	35-A	$M_{0.52}\frac{SO^{4}{}_{72}Cl_{21}}{{\left(Na+K\right)}_{100}}T22.8\;{}^{\circ}C$
WS 18		SO_4_-(Na+K)	35-A	$M_{0.49}\frac{SO^{4}{}_{66}Cl_{24}HCO^{3}{}_{11}}{{\left(Na+K\right)}_{100}}T22.9\;{}^{\circ}C$
WS 06	+347 m	HCO_3_·SO_4_-(Na+K)	14-A	$M_{0.81}\frac{HCO^{3}{}_{48}SO^{4}{}_{42}Cl_{10}}{{\left(Na+K\right)}_{99}}T23.5\;{}^{\circ}C$
WS 12		HCO_3_·SO_4_-(Na+K)	14-A	$M_{1.02}\frac{HCO^{3}{}_{63}SO^{4}{}_{29}}{{\left(Na+K\right)}_{99}}T22.4\;{}^{\circ}C$
WS 15		SO_4_·HCO_3_-(Na+K)	14-A	$M_{0.63}\frac{SO^{4}{}_{45}HCO^{3}{}_{41}Cl_{14}}{{\left(Na+K\right)}_{99}}T22.4\;{}^{\circ}C$
WS 19		HCO_3_·SO_4_-(Na+K)	14-A	$M_{0.65}\frac{HCO^{3}{}_{43}SO^{4}{}_{43}Cl_{14}}{{\left(Na+K\right)}_{100}}T22.6\;{}^{\circ}C$
WS 07	+355 m	SO_4_·HCO_3_-(Na+K)	14-A	$M_{0.81}\frac{SO^{4}{}_{46}HCO^{3}{}_{44}}{{\left(Na+K\right)}_{100}}T22.9\;{}^{\circ}C$
WS 10		HCO_3_·SO_4_-(Na+K)	14-A	$M_{1.05}\frac{HCO^{3}{}_{62}SO^{4}{}_{31}}{{\left(Na+K\right)}_{97}}T22.3\;{}^{\circ}C$
WS 16		HCO_3_·SO_4_-(Na+K)	14-A	$M_{0.76}\frac{HCO^{3}{}_{47}SO^{4}{}_{43}}{{\left(Na+K\right)}_{98}}T23.1\;{}^{\circ}C$
WS 20		HCO_3_·SO_4_-(Na+K)	14-A	$M_{1.03}\frac{HCO^{3}{}_{62}SO^{4}{}_{31}}{{\left(Na+K\right)}_{99}}T23.5\;{}^{\circ}C$
